# Interdisciplinary science, inspired by cephalopods

**DOI:** 10.1016/j.isci.2024.111315

**Published:** 2024-12-16

**Authors:** Robyn Crook, Alon A. Gorodetsky

**Affiliations:** 1 Department of Biology, San Francisco State University, San Francisco, CA 94132, USA; 2 Department of Chemical and Biomolecular Engineering, University of California, Irvine, Irvine, CA 92617, USA

## Abstract

Cephalopods, marine animals like squid, octopus, cuttlefish, or nautilus, have attracted the interest of the scientific community for many years due to their sophisticated neurophysiology, complex behavioral patterns, and stunning camouflage displays. It is thus not surprising that these animals have inspired a plethora of scientific advances in the fields of neuroscience, cell biology, and materials engineering. A part of this exciting research is reflected in the Special Issue “Cephalopods: Inspired science”.

## Main text

**Figure:**
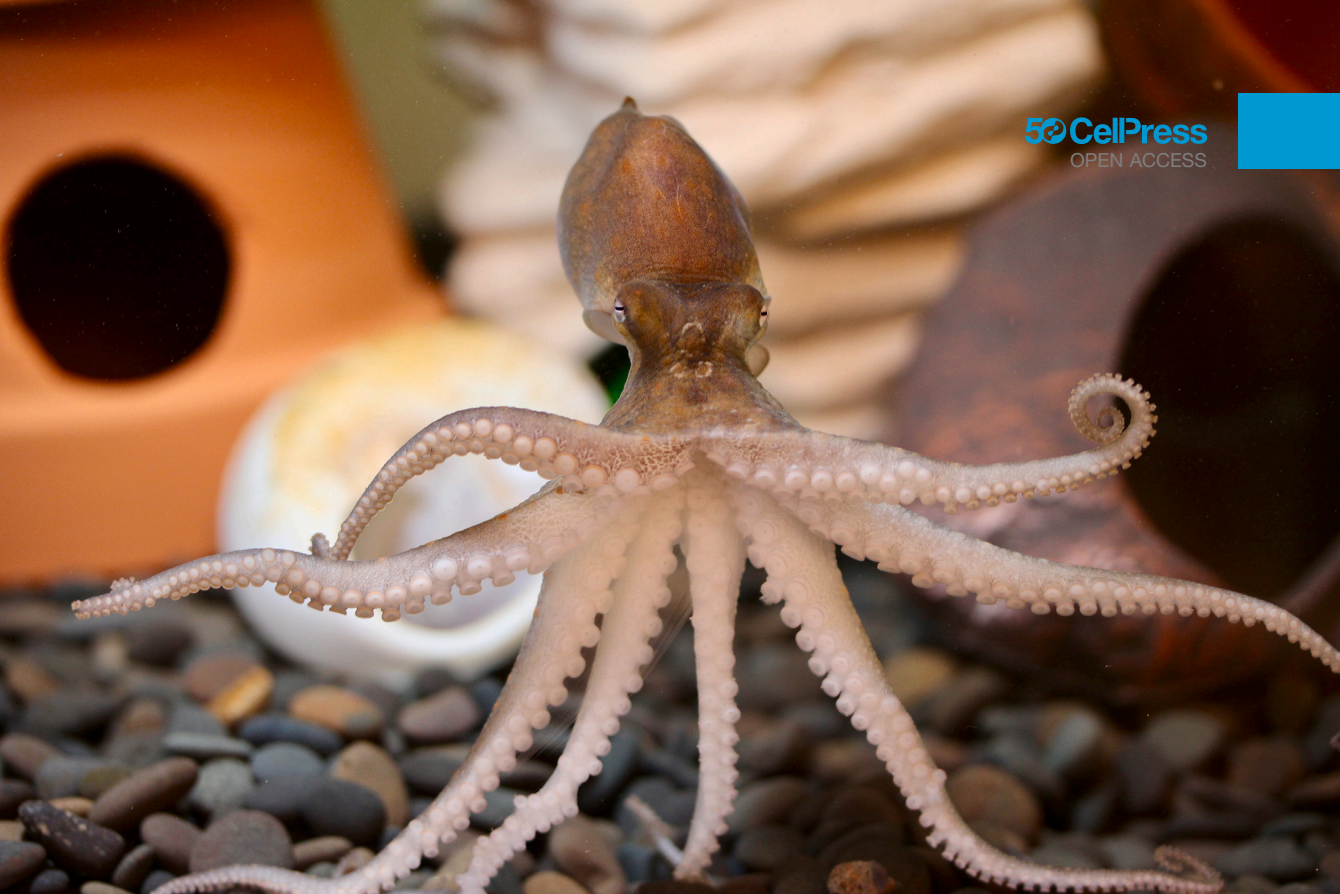
Above image: Image of a cephalopod (lab of Prof. Robyn Crook, San Francisco State University). Octopuses are valuable comparative models for neuroscience, cell biology, materials enginering, and many emerging fields.

I have found a lot of value in interacting with researchers that are quite far outside my own discipline. It has been a great experience overall, very invigorating and inspiring.

I would encourage young scientists to not be afraid to ask important questions and to take risks when trying to answer these questions

My hope is that by combining efforts across disciplines we can shed new light on the diversity of complex nervous systems and reveal fundamental principles of circuit design that thus far have really only been examined in the vertebrate lineage.

There is a big push towards recognizing their intelligence by treating these invertebrates in the same way as vertebrates from a regulatory perspective.

As part of the 50th anniversary of Cell Press in 2024, we are celebrating “science that inspires” and exploring the real-world impact of our science in meeting society’s greatest challenges. These complex challenges require interdisciplinary solutions and collaborative efforts. At *iScience*, we aim to promote and highlight this approach to scientific discovery, sharing stories focused on interdisciplinary research that stimulate discussion and celebrate the expanding diversity in scientific research. This Backstory is part of a series highlighting exciting researchers working in the fields of life, health, physical, and earth & environmental and social sciences who inspire with their interdisciplinary thinking while they work to meet society’s greatest challenges.

In this Backstory, the *iScience* editors discuss with the Guest Editors of the Special Issue “Cephalopods: Inspired science”, Prof. Alon Gorodetsky (from University of California, Irvine) and Prof. Robyn Crook (from San Francisco State University) who are sharing their thoughts about this topic, the current state of the field, the collection of articles in this Special Issue, the future of the research in this area in the coming years, and personal advice to aspiring young minds.

**Figure:**
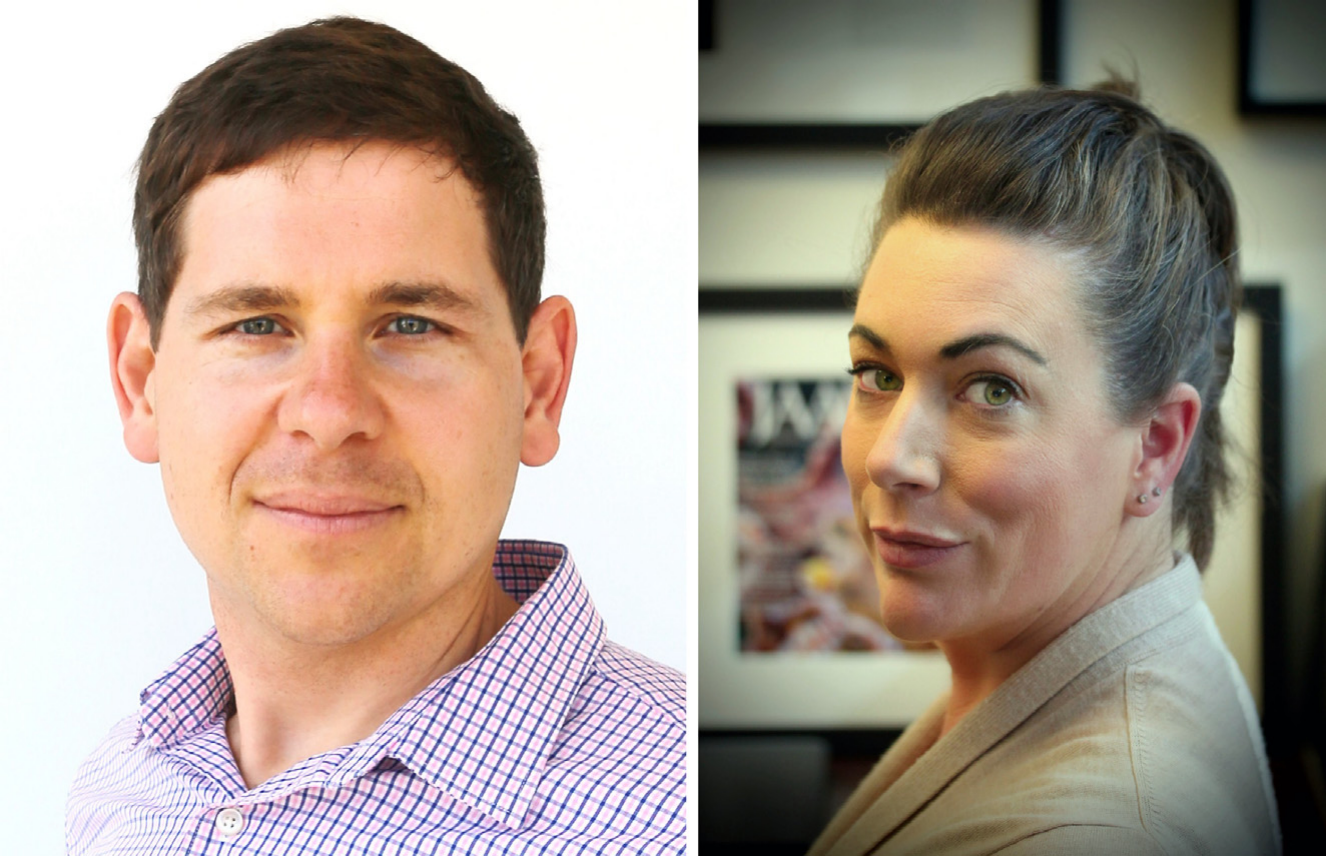
Left: Prof. Alon Gorodetsky (University of California, Irvine), Right: Prof. Robyn Crook (San Francisco State University).

### Introduction

#### What are your general background and interests? What originally interested you in the field?

**Alon Gorodetsky, University of California, Irvine**: My background is in Materials Science, Engineering Physics, and Chemistry. I was motivated to enter this field after attending a talk by Roger Hanlon from the *Marine Biological Laboratory in Woods Hole*, *MA*, who was showing incredible videos of octopus camouflage. I was hooked immediately!

**Robyn Crook, San Francisco State University**: My background is in marine biology and animal behavior, and I did not get interested in neuroscience until my postdoc. I have always been interested in evolutionary questions relating to behavior, which is where my interest in neural and behavioral responses to tissue injury originated.

#### Is your background and experience typical or similar to other researchers in the field? Why does a scientist choose to work in this field?

**Alon**: My background is not typical for researchers in this field, who often have more biology-focused expertise. My experience of entering the field is also unique because most people start at a much earlier stage in their careers in this area. Of course, a lot of scientists do enter the field because they enjoy spending time outdoors or near the ocean.

**Robyn**: Not really; most neuroscientists do not come from evolutionary biology or even from organismal biology. My belief is that broad perspectives on how behaviors are generated are critical for understanding the brain. Cephalopod neuroscience is explicitly evolutionary, yet there is still quite minimal focus on selection pressures and ecological factors when neuroscientists approach the brain of cephalopods.

### Interdisciplinarity and motivation

#### What role does interdisciplinarity play in your research?

**Alon**: Interdisciplinarity is very important in our work because we bring tools from chemistry, physics, and materials science to bear on understanding cephalopod camouflage. We have recently even further extended our toolkit to optical modeling. Our combined approaches are less common in the cephalopod community.

**Robyn**: We are in the early stages of connectomic studies on the octopus arm (*reconstructing the full organization of every cell and its connections within a nervous system*), so this has brought our lab into contact with computer scientists and machine learning experts in a new way. It has really broadened the scope of our work and the types of questions we can ask.

#### Does this interdisciplinarity create any barriers for entering this field, e.g., training? How could such barriers be overcome?

**Alon**: The major barriers that I have seen for entry are associated with terminology. For example, species names can change often and in unpredictable ways. Sometimes, I would give anything for footnotes or a glossary in some biology subdisciplines!

**Robyn**: Finding a common language among different disciplines can be a challenge. I would likewise be happy to have a glossary for some engineering terms!

#### What is the story behind this collaboration?

**Alon**: We were introduced for this project by the *iScience* editorial team. I knew Robyn by mutual connection and by reputation from having read some of her previous work at the *Marine Biological Laboratory in Woods Hole*, *MA*. It has been so much fun!!

**Robyn**: We were introduced for this project by the *iScience* editorial team. Although I knew Alon’s group’s work, we did not know each other personally before working together on this issue. It has been an enjoyable collaboration!

#### What has your experience been working in this interdisciplinary research and your participation/collaboration as Guest Editors of this special issue?

**Alon**: It has been a lot of fun to work on this issue! I have been able to broaden my horizons and understanding of cephalopod neurophysiology as a result.

**Robyn**: It has been so interesting to see the different types of research done outside the typical neuroscience and behavior where my lab normally focuses. I have found a lot of value in interacting with researchers that are quite far outside my own discipline. It has been a great experience overall, very invigorating and inspiring.

#### What is your opinion about the collection of articles in this special issue?

**Alon and Robyn**: We have a very nice mix of manuscripts that range from molecular-to cellular- to organismal-level understanding of cephalopod neurophysiology, with materials applications also thrown into the mix. We were both impressed by the quality, novelty, and depth of the articles.

### Future

#### What suggestions would you give to a young scientist interested in the field?

**Alon**: I would encourage young scientists to not be afraid to ask important questions and to take risks when trying to answer these questions. I would also encourage young scientists to get some hands-on experience with these amazing animals and their almost unbelievable abilities, which provides insight that cannot be easily gained from reading the literature.

**Robyn**: I think focusing on broad questions is more important than on techniques or taxa; technological advancements and tools are advancing so rapidly that you will often have to learn something new even when you are well established, but learning how to identify major outstanding questions in a field will always allow you to be doing interesting, important work. Ultimately, I would encourage anyone interested in cephalopod science to find a mentor doing exciting work, and then take as much time as possible learning about the biology of the animal before jumping into experimental research. Knowing about the natural behavior of cephalopods will help you find those big questions.

#### What are the most important/most exciting/interesting questions in the field currently, and why do they matter? What do you think are the biggest challenges that the field is facing?

**Alon**: Some of the most important and exciting questions revolve around understanding how cephalopods can nearly instantaneously perceive their surroundings in nature and then translate their perception of the surroundings to rapid skin color and appearance changes. The ability of their nervous systems to enable dynamic control over such effects is absolutely remarkable.

**Robyn**: There has been a resurgence of interest in spontaneous, unconstrained behavior and how neural circuits operate to optimize behavior in an animal’s natural environment. This is a hugely difficult task procedurally but also an area of great opportunity to really understand how the brain works. Cephalopods are ideal for this type of work but there are very substantial practical and ethical challenges.

#### What are other disciplines that should look to this field with interest? Which new communities do you see forming in the future around the topics the field is interested in?

**Alon**: Many emerging areas in engineering are looking to and benefiting from this field. These areas include robotics, sustainability, and adaptive optical materials. It seems that the optics and photonics communities are at the forefront of using cephalopods as inspiration. The way that cephalopods camouflage themselves is eye-catching and beyond what is possible with artificial materials!

**Robyn**: There is an emerging community focused on cephalopod neurophysiology, which is currently coalescing. This community is certain to grow in the future. I think there is a lot of value in comparative, non-traditional models for biomedical and human-centric neuroscience. A broader understanding of how different brains work would allow a new perspective on the human brain in health and disease.

#### What’s next? What breakthroughs do you imagine or hope to see in upcoming years?

**Alon**: I would hope to see a deeper understanding of the possibility that cephalopods perceive light with the entirety of their skin, which would also provide insight into how these animals control their amazing camouflage abilities. There is some exciting initial work along these lines, but a lot more exploration can be done.

**Robyn**: There is a strong push to create open data resources for cephalopods at the moment and I expect that this will lead to breakthroughs in understanding the structure and function of the nervous system, which until now has been very opaque and very challenging. My hope is that, by combining efforts across disciplines, we can shed new light on the diversity of complex nervous systems and reveal fundamental principles of circuit design that thus far have really only been examined in the vertebrate lineage.

### Conclusions and final thoughts

**Alon**: We both believe that cephalopods are far more intelligent than they are given credit for right now. There is a big push toward recognizing their intelligence by treating these invertebrates in the same way as vertebrates from a regulatory perspective.

**Robyn**: Cephalopods are amazing and intelligent animals who have a lot to offer to neurobiology and other fields. We have an enormous responsibility as we grow this field to ensure that the animals on which we work are treated ethically and carefully. There is growing, global recognition of sentience in invertebrates, including cephalopods, which means that newly developed research techniques and areas must take into account the realistic possibility that cephalopods can suffer and experience pain and distress. Every researcher working with cephalopods must take this possibility seriously.

